# PacBio Full-Length Transcriptome of a Tetraploid *Sinocyclocheilus multipunctatus* Provides Insights into the Evolution of Cavefish

**DOI:** 10.3390/ani13213399

**Published:** 2023-11-02

**Authors:** Renyi Zhang, Qian Duan, Qi Luo, Lei Deng

**Affiliations:** School of Life Sciences, Guizhou Normal University, Guiyang 550025, China; duanqian628@163.com (Q.D.); qiluo20@126.com (Q.L.); dengleimonkey520@163.com (L.D.)

**Keywords:** karst, cavefish, full-length transcriptome, phylogeny, evolution

## Abstract

**Simple Summary:**

As a national second-class animal in China, it is urgent to protect the wild population resources of *Sinocyclocheilus*. In this study, we constructed the first full-length transcriptome of *Sinocyclocheilus multipunctatus* from the molecular perspective, analyzed and inferred its phylogenetic relationships, divergence time, and whole-genome duplication events, and screened 220 positive selection genes involved in gene control, signal transduction, immune response, and other processes from the A- and B-subgenome of *S. multipunctatus*. This will provide basic support for future evolutionary and genomic studies on the cave adaptation mechanism of this species.

**Abstract:**

*Sinocyclocheilus multipunctatus* is a second-class nationally protected wild animal in China. As one of the cavefish, *S. multipunctatus* has strong adaptability to harsh subterranean environments. In this study, we used PacBio SMRT sequencing technology to generate a first representative full-length transcriptome for *S. multipunctatus*. Sequence clustering analysis obtained 232,126 full-length transcripts. Among all transcripts, 40,487 were annotated in public databases, while 70,300 microsatellites, 2384 transcription factors, and 16,321 long non-coding RNAs were identified. The phylogenetic tree showed that *S*. *multipunctatus* shows a closer relationship to *Carassius auratus* and *Cyprinus carpio,* phylogenetically diverging from the common ancestor ~14.74 million years ago (Mya). We also found that between 15.6 and 17.5 Mya, *S. multipunctatus* also experienced an additional whole-genome duplication (WGD) event, which may have promoted the species evolution of *S. multipunctatus*. Meanwhile, the overall rates of evolutionary of polyploid *S. multipunctatus* were significantly higher than those of the other cyprinids, and 220 positively selected genes (PSGs) were identified in two sub-genomes of *S. multipunctatus*. These PSGs are likely to fulfill critical roles in the process of adapting to diverse cave environments. This study has the potential to facilitate future investigations into the genomic characteristics of *S. multipunctatus* and provide valuable insights into revealing the evolutionary history of polyploid *S. multipunctatus*.

## 1. Introduction

Second-generation sequencing platforms represented by Roche 454, Illumina/Solexa, and ABI SOLiD are widely used in transcriptome sequencing because of the advantages of their short sequencing time, low cost, high accuracy, and high throughput [1]. However, because of the shorter read length of second-generation sequencing, it is difficult to obtain the full-length sequence information of genes without a reference genome. Third-generation sequencing technology developed in recent years has become a better choice, such as the single molecule real-time (SMRT) technology of the PacBio sequencing platform, which has become the primary choice for obtaining full-length transcription sequences [2]. The main advantage of third-generation sequencing technology is the long-fragment reads (average read length is up to 20 kb) [3]. The full-length transcript generated using reverse transcription does not need to be fragment processed, and the full-length sequence information can be obtained directly by single-molecule sequencing [4]. This technology has been extensively utilized in various aquatic animals, such as *Schizothorax lissolabiatus* [5], *Gymnocypris namensis* [6], *Schizothorax prenanti* [7].

The freshwater fish genus *Sinocyclocheilus* (Fang, 1936) (Cyprinidae: Barbinae) is an endemic allotetraploid fish species [8] in China, mainly distributed in karst areas of southwest China, including Yunnan Province, Guizhou Province, Guangxi Zhuang Autonomous Region, and Hubei Province [9]. They are a typical cave-restricted animal that lives in dark underground environments. Over time, they have evolved to adapt to the cave environments through a variety of characteristic changes in morphology, behavior, and physiology [10]. To date, there are 78 effective species in the *Sinocyclocheilus* genus [11], which is the largest group of cyprinid fish in China. Nine species of *Sinocyclocheilus* are listed on the IUCN Red List of Threatened Species (https://www.iucnredlist.org/, accessed on 30 September 2023), including two critically endangered (CR), one endangered (EN), and six vulnerable (VU). *Sinocyclocheilus multipunctatus* was originally named *Schizothorax multipunctatus* by Jacques Pellegrin in 1931 [12]. Subsequently, the species was placed in the genus *Sinocyclocheilus* and renamed *Sinocyclocheilus multipunctatus* [13]. *S*. *multipunctatus* is mainly distributed in the Guiyang and Qiannan Buyei and Miao autonomous prefectures in China. *S. multipunctatus* is especially threatened by overfishing, water pollution, and habitat loss due to its limited distribution [14]. Currently, the species is rated as a near-threatened species in the Red List of China’s Vertebrates [15].

In the current study, PacBio SMRT sequencing was used to generate the first full-length transcriptome of *S. multipunctatus*. Open reading frame (ORF) prediction and long non-coding RNA (lncRNA) identification, transcription factor (TF) prediction, and simple sequence repeat (SSR) analysis, as well as functional annotation and classification of transcripts were performed in this study. Comparative analyses with six species were conducted, focusing particularly on phylogenetic relationships, divergence time, and so on. Then, the *S. multipunctatus* whole-genome duplication (WGD) event was determined, and the evolution of positively selected genes in *S. multipunctatus* was analyzed. This study offers a valuable genetic repository of full-length transcripts, serving as a crucial resource for future studies of adaptive evolution, population genetics, and conservation in *S. multipunctatus*.

## 2. Materials and Methods

### 2.1. Sample Collection and RNA Preparation

**Figure 1:**
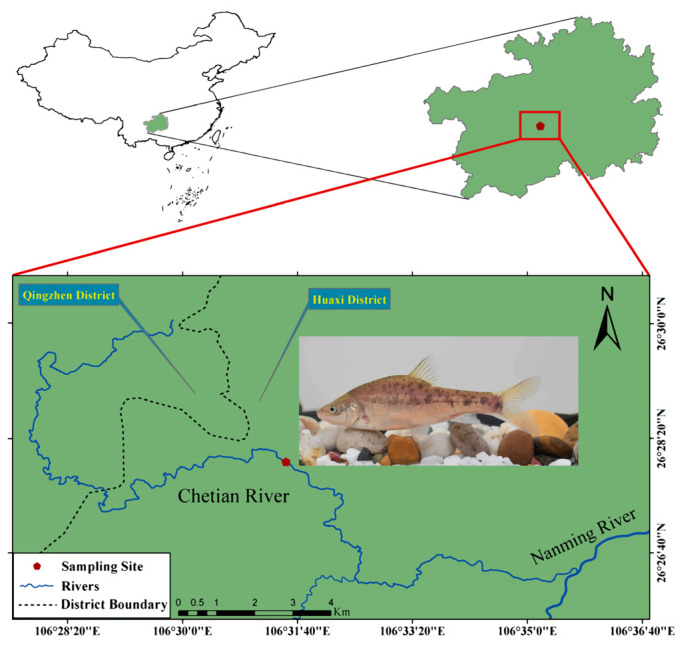
Sample collection sites for *S. multipunctatus*.

Three wild *S. multipunctatus* were collected in January 2022 from the Chetian River (Figure 1). Sampling was approved by the Department of Agriculture and Rural Affairs of Guizhou Province. After anesthesia with MS222, seven tissues including the brain, spleen, liver, kidney, gill, muscle, and skin were sampled. Equal amounts of 21 samples were pooled together and immediately stored in liquid nitrogen until RNA was extracted. The total RNA was isolated using TRIzol Reagent (Invitrogen, Waltham, MA, USA) according to the manufacturer’s instructions. RNA degradation and contamination were assessed on 1% agarose gels. The purity, concentration, and integrity of the RNA sample were assessed using the Nanodrop microspectrophotometer from Thermo Fisher Scientific (Waltham, MA, USA) and the Agilent Bioanalyzer 4200 system from Agilent Technologies (Santa Clara, CA, USA).

### 2.2. PacBio Iso-Seq Library Preparation and Sequencing

After RNA extraction, mRNA was enriched with Oligo (dT) microbeads. The SMARTer™ PCR cDNA Synthesis Kit (Clontech, Palo Alto, CA, USA) was used to synthesize full-length cDNA. Then, the cDNA was amplified by PCR. Amplified cDNA was purified using Pronex beads. The SMRTbell Template Prep Kit (Clontech, Palo Alto, CA, USA) was performed to repair and ligate the purified product. After exonuclease digestion, a single SMRT library was submitted to the PacBio Sequel platform for sequencing. All sequencing operations were conducted at the DNA Stories Bioinformatics Center (Chengdu, China).

### 2.3. PacBio Iso-Seq Data Processing

Sequence data were processed using the Iso-Seq3 v.4.0.0 (https://github.com/PacificBiosciences/IsoSeq3, accessed on 8 January 2023) software. Circular consensus sequences (CCS) were produced from subread BAM files using the parameters of min_length 10, min-passes 3, top-passes 60, min-rq 0.9, and max_length 50,000. The CCS were then processed using Lima (v. 2.2.0, https://lima.how/, accessed on 10 January 2023) for primer removal and demultiplexing. By searching for the presence of poly(A) signal and concatemers, full-length non-chimera (FLNC) reads were identified from CCS reads. The FLNC reads were then clustered into clusters by ‘isoseq3 cluster’ [16]. Unclustered singletons, with a quality score (Q20) of at least 99%, were retained and redundancy removal was performed with cd-hit-est. Finally, the final full-length transcripts were divided into either clusters or singletons. Next, to remove the redundancy, the final full-length transcripts were initially used as queries to TBLASTX [17] search reference genome *Cyprinus carpio* (GCF_018340385.1) and then collapsed transcripts by genomic mapping. The filtered alignments were clustered into independent genic loci.

### 2.4. Functional Annotation of Transcripts

The open reading frames (ORFs) were identified using TransDecoder (http://github.com/TransDecoder/TransDecoder, accessed on 12 January 2023) to obtain the coding sequences (CDS). Within a locus, the longest coding region was selected as representative of this isoform. When a locus was a noncoding region, only the longest transcript within a locus was retained for representative transcript. For functional annotation, the representative transcripts were subjected to similarity search against databases including NCBI non-redundant protein sequences (NR), Clusters of Orthologous Groups of proteins (KOG), Swiss-Prot, Gene Ontology (GO), Kyoto Encyclopedia of Genes and Genomes (KEGG), and Non-supervised Orthologous Groups (eggNOG) databases [18].

### 2.5. Gene Structure Analysis and Annotation

Based on the parameter of E -0.0001, the set of fish transcription factors (TFs) was identified using Animal TFDB v.2.0 [19]. The HMMER v.3.0 (http://hmmer.janelia.org/, accessed on 12 January 2023) algorithm was applied to distribute genes to different TF gene families. In order to predict a more complete SSR, transcripts with a length greater than 160 bp were selected for SSR analysis and identified by using GMATA software (https://sourceforge.net/projects/gmata/?source=navbar, accessed on 12 January 2023) [20]. A combination of four tools, including CNCI [21], PLEK [22], CPC2 [23], and CPAT [24] were used to search for lncRNA candidates from conjectured protein-coding RNAs.

### 2.6. Phylogenetic Analysis

Protein sequences of five species with genome available were downloaded and included in our analyses, including *Danio rerio* (GCF_000002035.6), *Megalobrama amblycephala* (GCF_018812025.1), *Ctenopharyngodon idellus* (GCGD: Grass Carp Genome Database), *Cyprinus carpio* (GCF_018340385.1), *Carassius auratus* (GCF_003368295.1). For the golden fish, the 1–25 chromosomes were assigned to A-subgenome, while the 26–50 chromosomes were assigned to B-subgenome. For the common carp (*Cyprinus carpio*), we used the A-subgenome and B-subgenome provided by the authors [25]. The A-subgenome and B-subgenome of *S. multipunctatus* were identified using the BLASTN program.

BLASTP [26] with an E-value of 1 × 10^−5^ was used for self-matching of the summary protein sequences of genomically available species, and low-quality fragments with identity <30% and coverage <30% were removed. Orthologous groups were established by orthofinder2 [27], which was with the default settings based on the screened BLASTP results. From the orthifinder2 results, we extracted single-copy gene families to obtain single-copy gene families among *S. multipunctatus* and the other five species. Then, from each single-copy gene family, the protein sequences were compared by using MUSCLE v. 3.8.31 [28] of the default parameters, and the homologous CDS alignments were back-translated from the homologous protein alignments using PAL2NAL [29]. The Gblocks [30] software (http://www.phylogeny.fr/one_task.cgi?task_type=gblocks, accessed on 12 January 2023) was applied to extract the conserved CDS alignments. The remaining CDS sequences of every single-copy family were used for further phylogenetic genomic analyses.

When the phylogenetic tree was constructed, CDS alignments of every single-copy family were concatenated to produce a super-matrix. RAxML [31] was used to construct the super-genes from the full-length and 4DTv sites, which performed to produce a maximum likelihood tree with the GTR + I + Γ model. The linked supergenes were divided into three regions corresponding to the 1st, 2nd, and 3rd codon sites in the CDS. Considering that the evolution rate of different codon positions is quite different, the three codon positions of the connected supergene were regarded as three distinct partitions. The MCMCTREE program in the PAML4.7 package [32] was supplied to estimate the divergence time under a relaxed clock model. The divergence times were calibrated with three calibrating points of zebrafish (*Danio rerio*) vs. common carp (*Cyprinus carpio*) ~55.8 Mya, grass carp (*Ctenopharyngodon idellus*) vs. common carp 20.5–20.95 Mya, and grass carp vs. *Megalobrama amblycephala* 9.1–22 Mya [33,34,35,36]. Based on the “Independent rates model (clock = 2)” and “JC69” model in MCMCTREE program, after the burn-in of 2,000,000 iterations, the MCMC process was run for 6,000,000 iterations. To confirm that the results were similar, each data type ran the program twice. FigTree v. 1.4.0 (http://tree.bio.ed.ac.uk/software/figtree, accessed on 12 January 2023) was used to produce the chronogram with the first run.

### 2.7. Detection of Polyploidization Events

The transcriptome sequences of *S. multipunctatus* were compared with BLASTP (E-value < 1 × 10^−5^) to find conserved paralog sequences to detect polypoidization events in the transcriptome of *S. multipunctatus*. Protein sequences of *Ctenopharyngodon idellus*, *Cyprinus carpio*, *Carassius auratus*, and three *Sinocyclocheilus* species were also analyzed and used for comparison using the WGDdetector software (https://github.com/yongzhiyang2012/wgddetector, accessed on 12 January 2023) [37], which has shown high performance in detecting recent and ancient WGD events; for example, it was applied in the study of WGD events of *Lautoconus ventricosus* [38], *Xenopus laevis* [37], and *Cyamopsis tetragonoloba* [39]. The protein and CDS sequences within each gene family were automatically compared using MAFFT [40] and PAL2NAL [29], and assigned the corresponding Ks values to each pair of similar sequences (gap-stripped comparison length > 90 bp) within each gene family based on the Nei–Gojobori algorithm. Then, the whole-genome duplication (WGD) events of each species were estimated based on the *Ks* distributions. The *Ks* values were transferred to the divergence time by the following formula T = *Ks*/2r, where r refers to the substitution rate used by *S*. *grahami* [41], 5.7–6.4 × 10^−9^ mutations per site per year [42].

### 2.8. Positive Selection Analysis

To estimate the branch-specific evolutionary rate for each species, the single-copy gene families generated by the Gblocks above were further used to explore the *Ka*, *Ks*, and *Ka*/*Ks* by running the free-ratio model (model = 1) on each orthologue through the CodeML program in the PAML package [43]. To obtain a reliable estimate, the estimated value on the branch was filtered. The filtering condition was the following: (1) synonymous sites < 1; (2) non-synonymous sites < 1; and (3) *Ks* ≥ 10. Then, we calculated the *Ka*/*Ks* for tandem sequences of all orthologs based on each orthologue and ten randomly selected homologues [44]. The Wilcoxon rank-sum test was used to identify GO categories with significantly higher *Ka*/*Ks* values, and the evolution rates of each lineage were compared [45]. The GO terms involving more than five orthologues were retained, the average *Ka*/*Ks* was calculated, the positive selection genes (PSGs) with *p*-values of less than 0.05 were screened out, and the lineage-specific accelerated GO categories were determined.

## 3. Results

### 3.1. Summary of FL Reference Transcriptome

In our study, 76,978,307 raw subreads were produced by using PacBio Sequel, with an average length of 1820 bp and an N50 length of 1957 bp (Table 1). A total of 1,776,276 CCS reads were obtained, with a mean length of 1950 bp. Subsequently, among these CCS reads, 1,554,240 full-length nonchimeric (FLNC) reads with an N50 length of 1959 bp were identified. Finally, a total of 232,126 full-length transcripts were obtained, with an average length of 2075 bp and an N50 length of 2338 bp, including 87,472 distinct clusters and 144,654 distinct singletons. The details are shown in Table 1. The sequences were mapped against the reference genome and generated 49,672 raw unique alignments.

### 3.2. Basic Annotation of Transcripts

**Figure 2:**
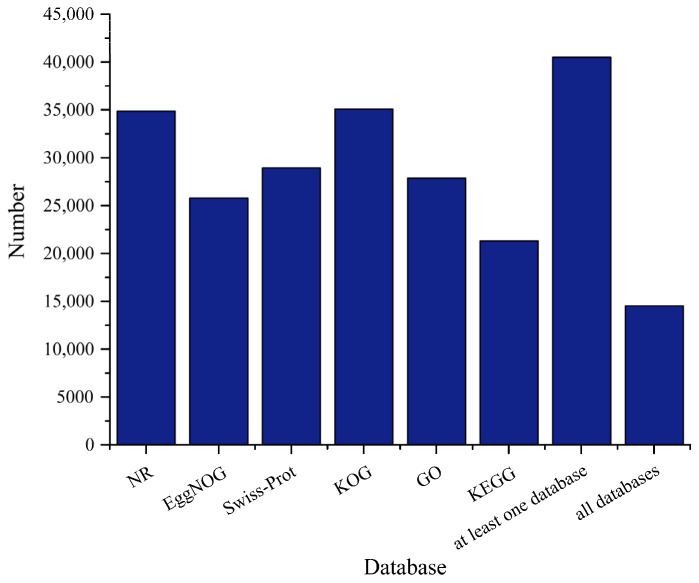
Statistics of the transcripts annotated in different databases.

In order to obtain comprehensive information on gene function, the representative transcripts were annotated by searching the NR, EggNOG, Swiss-Prot, KOG, GO, and KEGG databases. In total, 35,076 (70.62%) transcripts were annotated in the KOG; 27,879 (56.13%) were annotated in the GO; 22,599 (45.50%) in the KEGG; 28,940 (58.26%) in the Swiss-Prot; 26,175 (52.70%) in the EggNOG; and 34,861 (70.18%) in the NR (Figure 2). A total of 40,487 (81.51%) transcripts were annotated in at least one database, and 14,514 (29.22%) transcripts were annotated in all databases.

### 3.3. Detection of SSRs, TFs, and LncRNAs

**Figure 3:**
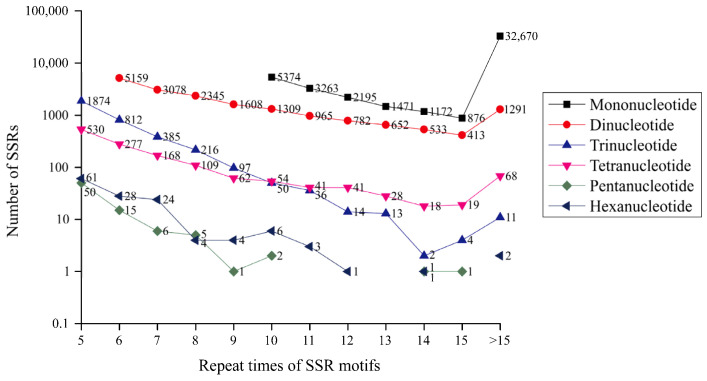
Summary of SSR types in the *S. multipunctatus* transcriptome. The X-coordinate is the repeat times of SSR motifs; the Y-coordinate is the number of SSR corresponding to the number of repeats of different repeat types. Different color shapes represent different types of SSRs.

**Figure 4:**
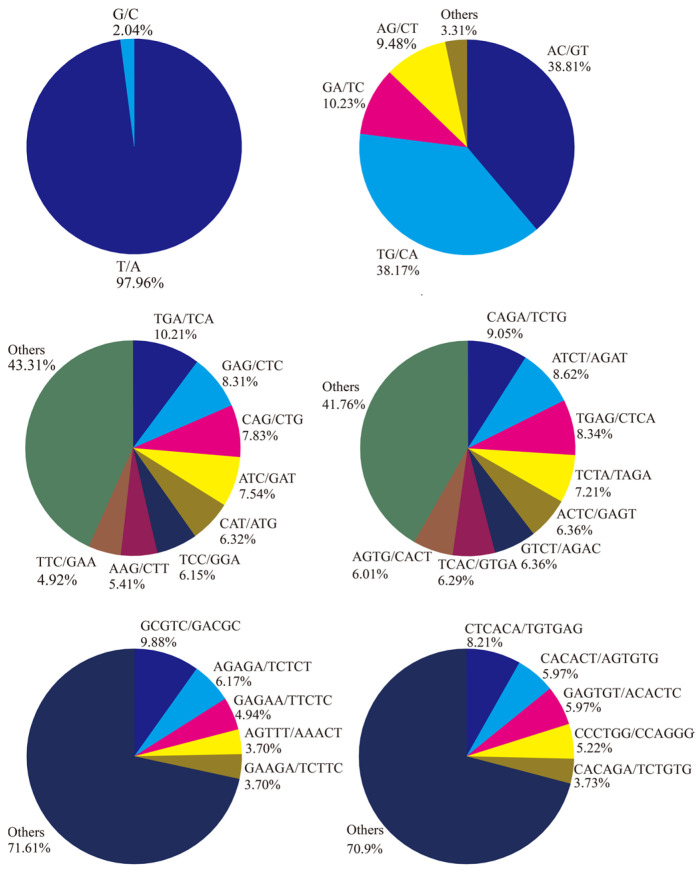
Statistics on the proportion of duplicate pairs of six types of nucleotides.

A total of 70,300 SSRs were identified from the total transcripts, of which 15,042 unique transcripts contained at least two SSRs. Mononucleotide was the leading repeat motif (47,021, 66.89%), followed by dinucleotide (18,135, 25.80%), trinucleotide (3514, 5.00%), and tetranucleotide (1415, 2.01%) (Figure 3). Only 81 (0.11%) and 134 (0.19%) SSRs were detected in pentanucleotide and hexanucleotide repeats, respectively. Among the mononucleotide SSRs, T/A accounted for 97.96% more than G/C (Figure 4). AC/GT (7039, 38.81%) was the richest motif in dinucleotide SSRs, followed by TG/CA (9926, 38.17%), and GA/TC (1855, 10.23%). For trinucleotide, tetranucleotide, pentanucleotide, and hexanucleotide SSRs, the most abundant motifs were TGA/TCA (359, 10.21%), CAGA/TCTG (128, 9.05%), GCGTC/GACGC (8, 9.88%) and CTCACA/TGTGAG (11, 8.21%), respectively (Figure 4).

**Figure 5:**
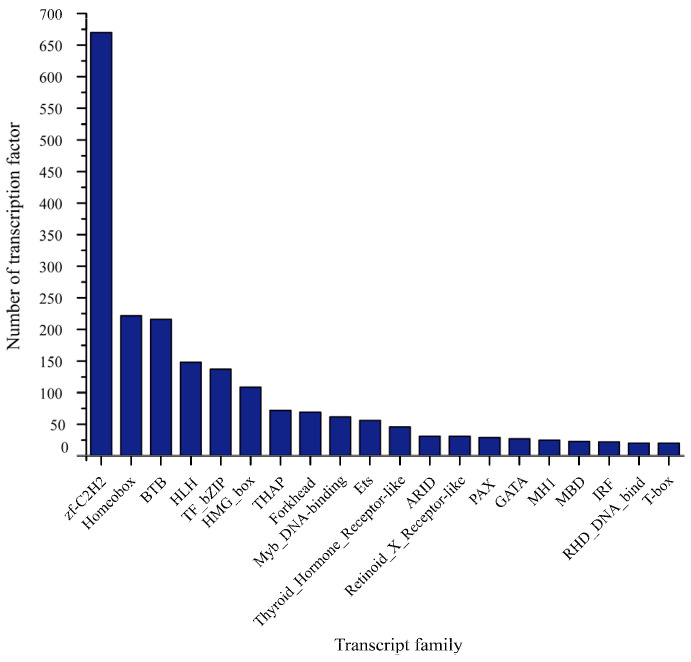
Identification of TFs in *S. multipunctatus*.

**Figure 6:**
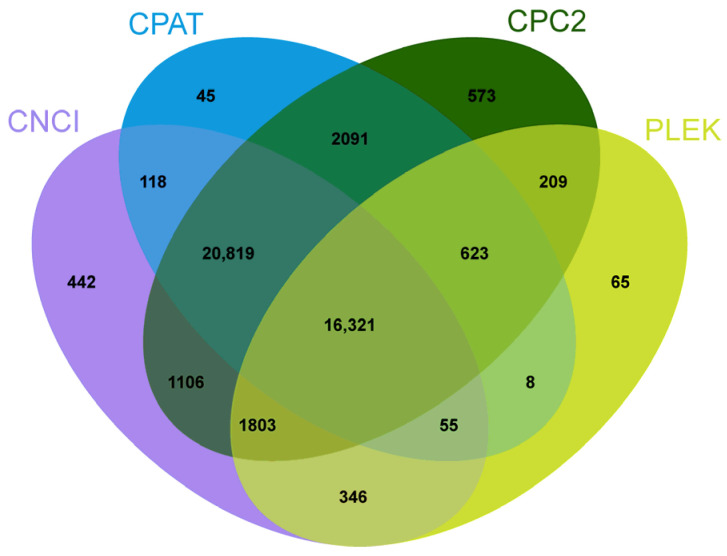
Venn diagram of lncRNAs predicted using CNCI, PFAM, PLEK, and CPC methods.

A total of 2384 TFs were identified, and the zf-C2H2 family (670) was the most represented, followed by the Homeobox family (222), BTB family (216), and HLH family (148) in our study (Figure 5). In total, 19,430, 41,010, 43,545 and 40,080 transcripts without protein-coding potential were identified by PLEK, CNCI, CPC2 and CPAT tools, respectively, and 16,321 overlapping transcripts were identified as assumed lncRNAs (Figure 6).

### 3.4. Identification of Orthologous Genes and Phylogenetic Tree

**Figure 7:**
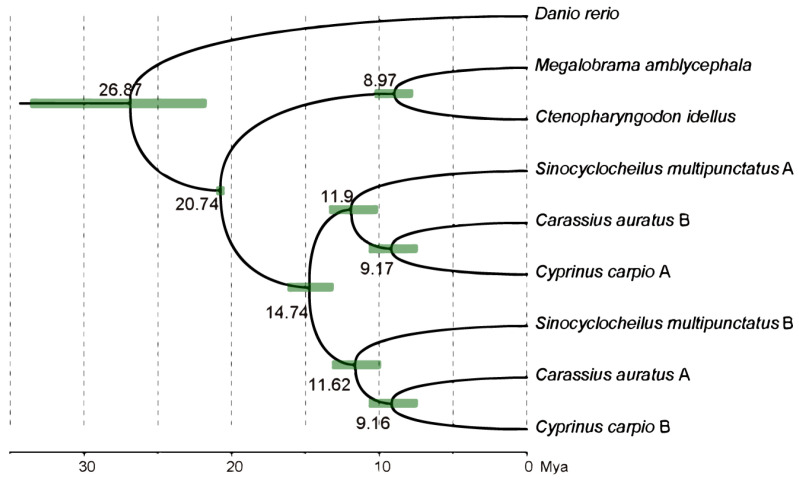
ML phylogenetic tree based on 2179 single-copy gene families of 6 fish species. The value at the nodes refers to the divergence time (Mya).

To obtain single-copy gene families for the *S. multipunctatus* and the other five species analyzed in this study, we first extracted 2280 single-copy gene families from the orthifinder2 results. The conserved CDS alignments were extracted by Gblocks, and a total of 2179 families remained. A total of 2179 single-copy gene families from six fish species were further identified to build a maximum-likelihood (ML) phylogenetic tree and calculated divergence time. The phylogenetic tree analysis revealed that *S*. *multipunctatus* had displayed a closer relationship to *Carassius auratus* and *Cyprinus carpio*; because there was no fossil correction, we based it on the three calibrating points, and surmised that *S*. *multipunctatus*, *Carassius auratus* and *Cyprinus carpio* had a phylogenetic divergence of common ancestors approximately 14.74 million years ago (Figure 7).

### 3.5. Genome Expansion in S. multipunctatus

**Figure 8:**
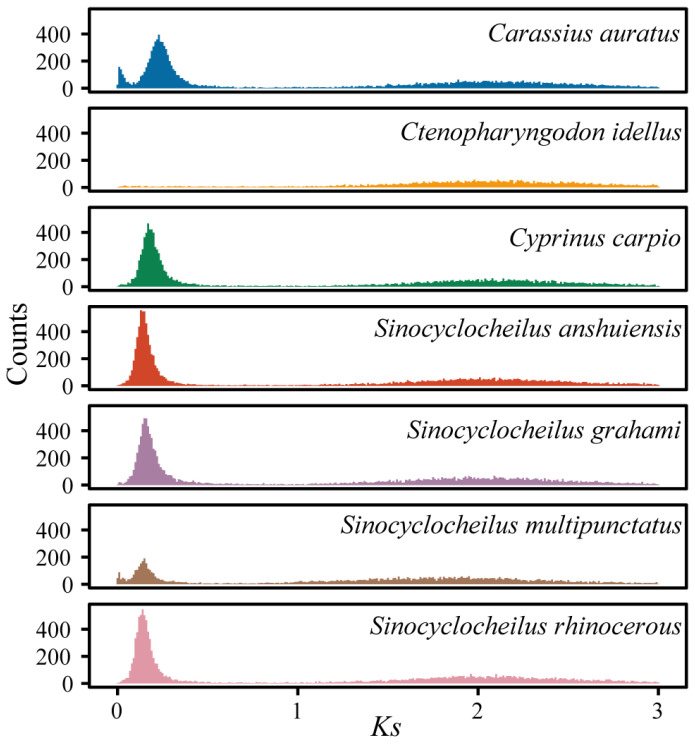
Whole-genome duplication events were detected in seven fish species. *X*-axis indicates the *Ks* (synonymous substitution rate) value of paralogous; *Y*-axis indicates the counts of paralogous gene pairs.

To research the genome expansion in *S. multipunctatus*, we analyzed whole-genome duplication (WGD) events. *Ks* values were estimated based on the homologous gene pairs from collinear regions of *S. multipunctatus* and six other representative cyprinid fish species. The distribution of *Ks* in *S. multipunctatus* showed one peak at *Ks* values of ~0.2 (Figure 8). Based on the reference nucleotide substitution rate, we estimated that the recent WGD event in *S. multipunctatus* was therefore estimated to occur about 15.6–17.5 million years ago (Mya). The peaks of the four *Sinocyclocheilus* fishes were very close to those of *Cyprinus carpio*, which means the four fish species may have shared the recent genome-wide duplication events.

### 3.6. Accelerated Evolution and Positively Selective Genes in S. multipunctatus

**Figure 9:**
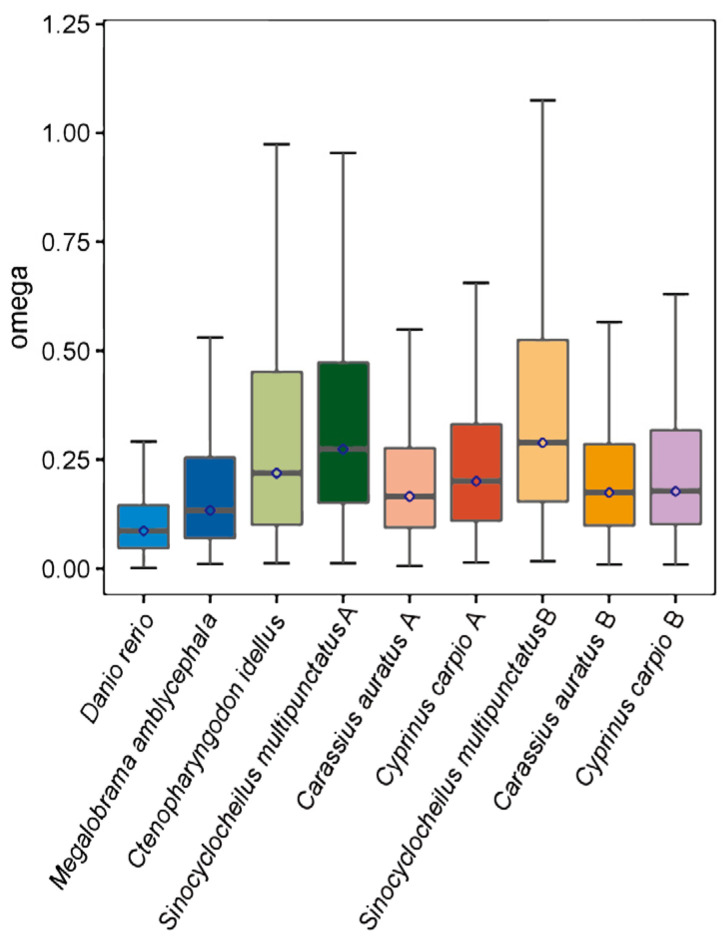
The *Ka*/*Ks* ratios (omega) of six species were calculated with the free-ratio model in CodeML program.

To evaluate the overall evolutionary rates based on concatenated alignments of all orthologues, we employed CodeML to calculate substitution rates (*Ka* and *Ks*) for each orthologue based on the use of the free ratio model. The *Ka*/*Ks* ratio level revealed the *Ka*/*Ks* ratio of the B-subgenome of *S. multipunctatus* lineage was higher than the other species (Figure 9), indicating that *S. multipunctatus* are evolving at a faster rate than other cyprinid species.

**Figure 10:**
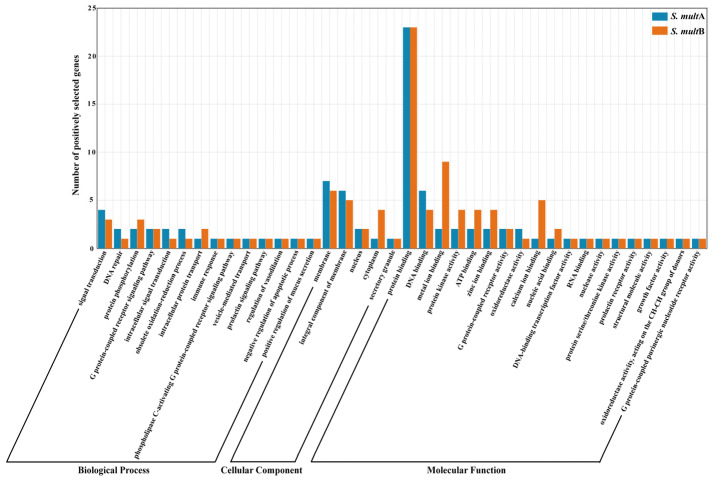
GO term annotation distributions of the positively selected genes (PSGs) in *S. multipunctatus* A-subgenome and B-subgenome in the same GO classification. *S. mult*A: the A-subgenome of *S. multipunctatus*; *S. mult*B: the B-subgenome of *S. multipunctatus*.

We used this model in CodeML to obtain the positively selected genes (PSGs) in codons along the *S. multipunctatus* sequences. A total of 220 PSGs were identified in the A-subgenome and B-subgenome of *S. multipunctatus*, and the GO functional annotation analysis showed that there were PSGs in all three GO terms. Meanwhile, the number of PSGs in the B-subgenome was more than that in the A-subgenome, while the number of PSGs in the cellular component (CC) was less than that in biological process (BP) and molecular function (MF) (Table S1). We classified the PSGs in the A-subgenome and B-subgenome of *S. multipunctatus* by GO categories and drew the distribution of GO term classifications of PSGs among the same GO categories (Figure 10). GO categories “signal transduction”, “DNA repair” and “protein phosphorylation” (in BP); “membrane”, “integral component of membrane” and “cytoplasm” (in CC); and “protein binding”, “DNA binding” and “metal ion binding” (in MF) contained the largest percentage of genes. In addition, the subgenomes A and B of *S. multipunctatus* also have different GO term descriptions, such as, “regulation of DNA-templated transcription” (in BP) and “sequence-specific DNA binding” (in MF), and were significantly enriched in A-subgenome, and these PSGs of B-subgenome were significantly enriched in “proteolysis” (in BP), “extracellular region” (in CC) and “cysteine-type peptidase activity” (in MF) (Table S1). These GO term descriptions indicated that the PSGs play key roles in the biological functions and environmental adaptations of *S. multipunctatus*.

## 4. Discussion

### 4.1. Long-Read Reference Reconstruction of the Full-Length Transcripts

*Sinocyclocheilus multipunctatus* is a unique fish in subterranean karst caves, but its genetic background, genetic diversity, and cave adaptability are still unknown. In this study, we first analyzed the full-length transcriptome of *S. multipunctatus*, and employed PacBio SMRT sequencing to produce 259 Gb clean data, including 1,776,276 CCS and 1,554,240 FLNC reads. After removing redundant sequences, 232,126 high-quality non-redundant full-length transcripts for *S. multipunctatus* were obtained. A total of 70,300 SSRs and 2384 TFs were identified. A total of 16,321 lncRNAs were predicted. Functional annotation of transcripts indicated that 40,487 transcripts were annotated into at least one functional database, much higher than no homologous sequences in the public databases. These consequences indicated that the integrality and quality of the full-length transcriptome obtained using SMRT sequencing is quite reliable, which can be used as a preliminary reference for future *Sinocyclocheilus* genome assembly and gene annotation.

According to previous studies, lncRNAs play key regulatory roles in important biological processes, such as gender regulation and aging, cell cycle and differentiation, and genetic regulation [46]. With the development of science and technology, a large number of studies have shown that lncRNA plays an increasingly important role in the regulation of epigenetics [47]. In this study, we identified a total of 16,321 lncRNAs on the non-redundant full-length transcript sequences, and found a large number of new lncRNAs in *S. multipunctatus*. Whether this is related to the adaptation of *S. multipunctatus* to the cave environment and its special morphological characteristics, and the function of lncRNAs still needs to be investigated in future studies.

It was found that TFs bind specifically to the regulatory regions of the genome through a sequence and play an important role in regulating gene transcription [48]. In that recent study, 2384 TFs from 68 families were projected in total, including zf-C2H2, Homeobox, BTB, HLH and others. Among them, zf-C2H2 occupied the largest proportion; it, as a member of the zinc-finger protein family, can recognize DNA, RNA, proteins or lipids by binding with metalions, thus regulating the expression of a large number of functional genes [49]. This will provide a useful reference for future research on the regulatory mechanisms of transcription factors in biological processes.

SSRs are short tandem repeats consisting of short tandem arrays of 1–6 base pairs, also known as microsatellites [50]. Furthermore, the identified SSR loci can be used for subsequent genetic and molecular marker-related studies [7]. Here, a total of 70,300 SSRs were identified in all full-length transcripts; the most abundant loci in *S. multipunctatus* were mono-nucleotides (A/T) and di-nucleotides (AC/GT, TG/CA), and this result is similar to that of the analysis of *Schizothorax prenanti*, *Nibea albiflora*, and *Squaliobarbus curriculus* [7,51,52]. This provided valuable future resources for marker-assisted breeding.

In summary, these full-length transcripts acquired in this study will lay a foundation for further research on the genetics and evolution of *S. multipunctatus* and other endangered *Sinocyclocheilus*.

### 4.2. Evolutionary Status and Positive Selection

In recent years, the universal ploidy variation of polyploidy has become a research hotspot, but the complexity of ploidy presents many challenges to the genetic or genomic research of polyploidy [5,53]. A previous study has shown that *Sinocyclocheilus* species have the closest phylogenetic relationship to *Cyprinus carpio* and *Carassius auratus* at the genomic level [54]. Based on one-to-one orthologous genes, *S. multipunctatus* had the closest evolutionary relationship with *Cyprinus carpio* and *Carassius auratus* when compared to *Danio rerio* and *Ctenopharyngodon idellus*, which coincides with the results inferred from genome data.

Positive selection, which focuses on adaptive evolution, is a significant source of species evolution and a major force behind species differentiation [55], during biological evolution. Here, we identified 220 candidate homologous genes that underwent positive selection in *S. multipunctatus*, and the mean and peak of *Ka*/*Ks* values of *S. multipunctatus* show in Figure 9 were higher than that of other fishes. This indicated that accelerated evolution occurred in *S. multipunctatus* after splitting from *Carassius auratus* and *Cyprinus carpio*. Accelerated evolution may be associated with the adaptation of *Sinocyclocheilus* fish to their unique cave environment. In addition, out of the six species, the overall rate of evolution of polyploid individuals was relatively faster compared to those of diploid species, which was similar to *Misgurnus anguillicaudatus* [56], showing that the evolutionary pressure of polyploid *S. multipunctatus* was increasing.

Positive selection analysis can identify genes that are related to functional and environmental change [57]. In order to define and describe the function of the 220 PSGs, GO functional annotation was statistically performed. The GO function classifications of PSGs identified from the *S. multipunctatus* clades revealed significant positive selective enrichment in biological processes and molecular function, particularly in gene control and cellular process. Notably, 23 PSGs of *S. multipunctatus* were involved in “protein binding” (GO:0005515); meanwhile, the genes, which are involved in DNA binding, signal transduction, DNA repair, and immune response, were positively selected in *S. multipunctatus*. This implies that these genes may be involved in the adaption process of *S. multipunctatus* to cave dwelling. In addition, in the cellular component, most of the PSGs are concentrated in the membrane structure, nucleus, and cytoplasm, which may indicate that most of the gene products play a positive selection to promote biological evolution throughout the cell during the adaptation of cavefish to the extreme cave environment.

### 4.3. The Whole-Genome Duplication Event in S. multipunctatus

All teleost fishes are generally believed to be undergoing third-round WGD (3R WGD, which also means the teleost-specific WGD) [58], and Cyprinidae fishes have experienced a recent whole-genome duplication event (thus 4R WGD) [59]. We performed *Ks* analyses to estimate the timing of occurrence of recent lineage-specific WGD in *S. multipunctatus*. The WGDs were predicted to occur around 15.6–17.5 Mya but the estimated times of the recent divergences were approximately ~14.74 Mya (Figure 7), indicating that whole-genome duplication events provide conditions for species divergence during species evolution. During geology and climate change, their divergence may be because of the geographical isolation formed by the continuous uplift of the Yunnan–Guizhou Plateau after Himalayan orogeny (40–50 Mya) [60], and some of their ancestral individuals may have swam down along the underground rivers into surrounding caves or dragon pools. Furthermore, the nearly overlap of the peak values indicated that *S. multipunctatus* and *Cyprinus carpio* might have shared the recent specific WGD together (Figure 8). In previous studies based on common carp (*Cyprinus carpio*), the time estimation of the latest WGD for *Cyprinidae* fishes ranging from 8.2–16 Mya was controversial [36,61,62]. However, with the development of fish genome research, a recent study in common carp established a general time range (9.7–23 Mya) and further predicted this time point to be about 12.4 Mya [63]. Our result of 15.6–17.5 Mya in the present study falls within the same time range, and the *Ks* analyses of *S. multipunctatus* will provide more evidence for the timing of recent genome duplication in *Cyprinidae*.

## 5. Conclusions

PacBio SMRT sequencing was used to gain the first comprehensive full-length transcriptome of *S. multipunctatus*, whose genome is not available. The acquisition of full-length transcripts makes gene annotation, the development of a molecular marker, and lncRNA prediction more accurate and reliable. Therefore, this study of the comprehensive full-length transcriptome of *S. multipunctatus* will provide an important resource for future research on functional genes, molecular markers, molecular events, and signaling pathways. Through a comparative analysis of phylogenetic relationships, divergence time, positive selection, and whole-genome duplication-event analysis, we can further understand the origin and speciation, as well as species polyploidization of *Sinocyclocheilus* fishes. Finally, this study will offer valuable support for future evolutionary and genomic research on the mechanisms underlying cave adaptability in this particular species.

## Figures and Tables

**Table 1:** Summary of the transcriptome of *S. multipunctatus* using the PacBio Sequel platform.

Types	Items	Number
Subreads	Subreads base (G)	259
	Number of subreads	76,978,307
	Average length (bp)	1820
	N50 length (bp)	1957
CCS reads	Number of reads	1,776,276
	Average length (bp)	1950
	N50 length (bp)	2081
FLNC reads	Number of reads	1,554,240
	Average length (bp)	1827
	N50 length (bp)	1959
Full-length transcriptome	transcripts number	232,126
	Average length (bp)	2075
	N50 length (bp)	2338

## Data Availability

The raw sequence data obtained in this study were submitted to the NCBI Sequence Read Archive (SRA) under the BioProject number PRJNA1021274, the BioSample number SAMN37549917, and the SRA accession number SRR26284563.

## Supplementary Materials

The following supporting information can be downloaded at: https://www.mdpi.com/article/10.3390/ani13213399/s1, Table S1: description of all positive selection genes in *S. multipunctatus* A- and B-subgenomes in GO functional annotation.
